# Prevalence and Factors Associated with Metabolic Syndrome among Non-Diabetic Saudi Adults: A Cross-Sectional Study

**DOI:** 10.3390/biomedicines11123242

**Published:** 2023-12-07

**Authors:** Basmah Eldakhakhny, Sumia Enani, Hanan Jambi, Ghada Ajabnoor, Jawaher Al-Ahmadi, Rajaa Al-Raddadi, Lubna Alsheikh, Wesam H. Abdulaal, Hoda Gad, Anwar Borai, Suhad Bahijri, Jaakko Tuomilehto

**Affiliations:** 1 Department of Clinical Biochemistry, Faculty of Medicine, King Abdulaziz University, Jeddah 22252, Saudi Arabia; gajabnour@kau.edu.sa (G.A.); hjad@kau.edu.sa (H.G.); sb@kau.edu.sa (S.B.); 2 Saudi Diabetes Research Group, King Fahd Medical Research Center, King Abdulaziz University, Jeddah 22252, Saudi Arabia; senani@kau.edu.sa (S.E.); hjambi@kau.edu.sa (H.J.); jalahamade@kau.edu.sa (J.A.-A.); rmsalharbi@kau.edu.sa (R.A.-R.); boraiaa@ngha.med.sa (A.B.); jotuomilehto@gmail.com (J.T.); 3 Food, Nutrition, and Lifestyle Research Unit, King Fahd for Medical Research Center, King Abdulaziz University, Jeddah 22252, Saudi Arabia; 4 Department of Food and Nutrition, Faculty of Human Sciences and Design, King Abdulaziz University, Jeddah 22252, Saudi Arabia; 5 Department of Family Medicine, Faculty of Medicine, King Abdulaziz University, Jeddah 22252, Saudi Arabia; 6 Department of Community Medicine, Faculty of Medicine, King Abdulaziz University, Jeddah 22252, Saudi Arabia; 7 Department of Biochemistry, Faculty of Sciences, King Abdulaziz University, Jeddah 21589, Saudi Arabia; lloo86@hotmail.com (L.A.); whabdulaal@kau.edu.sa (W.H.A.); 8 Medical Biochemistry and Molecular Biology Department, Faculty of Medicine, Alexandria University, Alexandria 21561, Egypt; 9 King Abdullah International Medical Research Center, King Saud Bin Abdulaziz University for Health Sciences, King Abdulaziz Medical City, Jeddah 22384, Saudi Arabia; 10 Department of Public Health, University of Helsinki, FI-00014 Helsinki, Finland; 11 Public Health Promotion Unit, Finnish Institute for Health and Welfare, FI-00271 Helsinki, Finland

**Keywords:** metabolic syndrome, type 2 diabetes, BMI

## Abstract

(1) Introduction: given the high prevalence of metabolic syndrome (MetS) in Saudi Arabia, especially in Jeddah, this study aims to understand the dietary and lifestyle-related risk factors among Jeddah’s non-diabetic adults. (2) Material and Methods: Employing a cross-sectional design, non-diabetic adults were sourced from public healthcare centers. Demographics, lifestyle, and dietary habits were surveyed. Blood pressure, anthropometrics, and fasting blood samples measuring plasma glucose, serum triglycerides, and HDL cholesterol were collected. The age cut-off for MetS was ascertained using the receiver operating characteristic curve. Variables influencing MetS were evaluated using univariate logistic regression, and consequential factors underwent multivariate analysis, adjusted for age and sex. (3) Results: Among 1339 participants, 16% had MetS, with age being the strongest predictor (*p* < 0.001). The optimal age cut-off was 32 years. For those <32, elevated BP in men and waist circumference (WC) in women were most prevalent. For those >32, elevated WC was dominant in both sexes. Univariate logistic regression revealed that higher income and education correlated with lower MetS prevalence, while marriage and smoking were risk factors. Adjusting for age and sex, only very high income had a significant low-risk association (*p* = 0.034). (4) Conclusion: MetS is notable in the studied group, with age as the pivotal predictor. High income reduces MetS risk, while marital status and smoking could increase it. Since this was a cross-sectional study, cohort studies are needed to validate our findings.

## 1. Introduction

The clustering of visceral obesity, hypertension, insulin resistance, and dyslipidemia, which is termed metabolic syndrome (MetS), is reported to increase the risk of developing type 2 diabetes (T2DM) and cardiovascular disease (CVD) [1,2,3]. Indeed, MetS is reported to increase the risk of developing T2DM by five-fold [4].

Several major organizations unified the diagnostic criteria for MetS, stating that the presence of any three of five risk factors (elevated blood pressure, elevated serum triglycerides, abdominal obesity, reduced high-density lipoprotein cholesterol (HDL-C), and elevated fasting glucose) indicates its presence, with specific cut-off values for each of these risk factors being defined and taking into account ethnic variations in the definition of abdominal obesity [5,6].

The prevalence and characteristics of MetS based on different definitions have been conducted over the years on different population groups in Saudi Arabia [7,8,9,10,11,12,13]. In a cross-sectional study conducted by the Saudi diabetes research group in the city of Jeddah [14], the prevalence of MetS in apparently non-hypertensive, non-diabetic individuals aged 18–50 years was 18.9% using the International Diabetes Federation (IDF) definition [14,15], 16.7% using the NCEP-ATP III definition [16], and 21.9% [14] using the most recent consensus definition [15].

The prevalence of MetS is influenced by genetic factors and metabolic susceptibility [17]; hence, it is population-specific and has been increasing globally due to rapid changes in available nutrition, lifestyle, and exposure to stress [17].

This rise of MetS is expected to lead to an increased risk of T2DM and CVD [18,19]. Diabetes is a costly medical disorder because of its associated chronic complications that can overburden health resources. Indeed, the medical cost for people with diabetes is 2.4 times compared to that for people without the condition, with chronic CVD and renal complications representing the costliest [20,21].

Due to the growth and prevalence of type 2 diabetes mellitus in Saudi Arabia [22,23], as well as the fact that the presence of MetS increases the risk of T2DM, it is imperative to try and prevent or manage MetS, especially since MetS can be managed by dietary and other lifestyle modifications [24,25,26]. Early detection of population-specific risk factors associated with MetS is imperative in order to initiate interventional health programs for management to prevent or delay the progression to more serious conditions [27,28]. There are no available data on the Saudi population’s dietary and lifestyle factors associated with MetS. Hence, we aimed to investigate the dietary and other lifestyle practices associated with increased risk of MetS in apparently healthy Saudi adults.

## 2. Materials and Methods

### 2.1. Study Design and Data Collection

This study focused on adult residents (aged 18 years and above) of Jeddah, who have not been diagnosed with diabetes. Our methodology, as previously detailed [29], guided the inclusion of participants. We excluded individuals with a history of cancer, renal or liver disease, cardiovascular diseases (CVDs), gastrointestinal disorders requiring specialized diets, physical or mental impairments, and pregnant women. The research protocol received approval from both the “Committee on Ethics of Human Research” at the Faculty of Medicine, King Abdulaziz University (KAU), and the “Committee on Research Ethics” at the Ministry of Health in the Kingdom of Saudi Arabia. The study employed a cross-sectional approach, enlisting 1500 participants, evenly split between men and women (750 each). This participant group was selected from various public healthcare centers across Jeddah. The selection process utilized a stratified, two-stage cluster sampling technique, ensuring representation from all health sectors in the city [30]. A consent form was signed by the recruited participants. Demographics, lifestyle variables, dietary habits, and personal medical and family history were obtained using a predesigned questionnaire based on factors associated with dysglycemia and other metabolic abnormalities found in other populations [31,32,33,34,35,36].

Participants were advised to undergo a fasting period of 10 to 14 h overnight. Following this fasting period, blood samples were taken to determine levels of fasting plasma glucose (FPG), as well as serum triglycerides (TG) and high-density lipoprotein cholesterol (HDL-c).

### 2.2. Anthropometric Indices

Measurements of various anthropometric parameters, including height, weight, waist circumference (WC), hip circumference (HC), neck circumference (NC), and body fat percentage (fat%), were conducted using standardized methods and equipment as previously described in our methodology [29]. Participants were measured for height without shoes, rounded to the nearest 0.5 cm using a fixed stadiometer. Weight was recorded, with participants in light clothing, to the nearest 0.5 kg using a calibrated portable scale (Omron BF511; OMRON Healthcare, Kyoto, Japan). WC was measured at the midpoint between the lowest rib and the iliac crest to the nearest 0.5 cm. These weight and height measurements were then used to calculate the body mass index (BMI), expressed in kg/m^2^. Blood pressure (BP) was also measured as part of this process.

### 2.3. Biochemical Assays

All collected samples, including whole blood, serum, and plasma, were processed and analyzed at the Clinical Chemistry Laboratory of the National Guard Hospital in Jeddah. The serum total cholesterol (TC), HDL-c, triglycerides (TG), and plasma glucose measurements were conducted using spectrophotometric techniques. These were performed in accordance with the provided guidelines using the Architect c8000 auto-analyzer (manufactured by Abbot, Abbott Park, IL, USA). The glycated hemoglobin (HbA1c) levels were determined using the HbA1c analyzer G8 from TOSOH Corporation, Tokyo, Japan. Additionally, a separate sample was taken one hour after the participants consumed a 50 g glucose solution (sourced from CASCO NERL Diagnostics, East Providence, RI, USA) to evaluate plasma glucose levels for the 1 h oral glucose challenge test (1h-OGTT) [37,38].

### 2.4. Definition of MetS

Participants were identified as having metabolic syndrome (MetS) if they exhibited three or more of the following criteria: waist circumference indicating normal adiposity (not exceeding 94 cm for men and 80 cm for women), elevated triglycerides (TG) marked by levels at or above 1.7 mmol/L (150 mg/dL) or if they were under treatment for hyperlipidemia; reduced HDL cholesterol (HDL-c) levels defined as below 1.0 mmol/L (40 mg/dL) for men and below 1.3 mmol/L (50 mg/dL) for women, or if they were receiving treatment for hyperlipidemia; high blood pressure (BP) characterized as systolic blood pressure (SBP) of 130 mmHg or higher, and/or diastolic blood pressure (DBP) of 85 mmHg or higher, or if they were on medication for BP reduction; and elevated fasting plasma glucose (FPG) levels, identified as FPG equal to or greater than 5.5 mmol/L (100 mg/dL), or if they were using glucose-lowering drugs [5].

### 2.5. Statistical Analysis

Data analysis was performed using IBM SPSS statistics version 26.0 for Windows. Baseline characteristics were expressed as frequencies and mean ± standard deviations (SDs). Demographic, clinical, dietary, and lifestyle variables of people with MetS were analyzed by comparing them to those without MetS. Independent *t*-test was used to compare factors with continuous variables between the two groups, while chi-square test or Fisher’s exact test, as appropriate, was used to compare categorical variables. The receiver operating characteristic (ROC) curve and the area under the receiver curve (AUC) were used to determine the age cut-off value for MetS. Unadjusted univariate logistic regression was used to assess the association between demographic, lifestyle, dietary, and outcome variables, namely MetS. Then, significant factors were included in a multivariate logistic regression model with adjustments for age and sex to explore the independent effects on MetS, and the adjusted prevalence ratios (PRs) were calculated. A *p*-value less than 0.05 (using a two-sided test) was considered to indicate statistical significance.

## 3. Results

**Figure 1:**
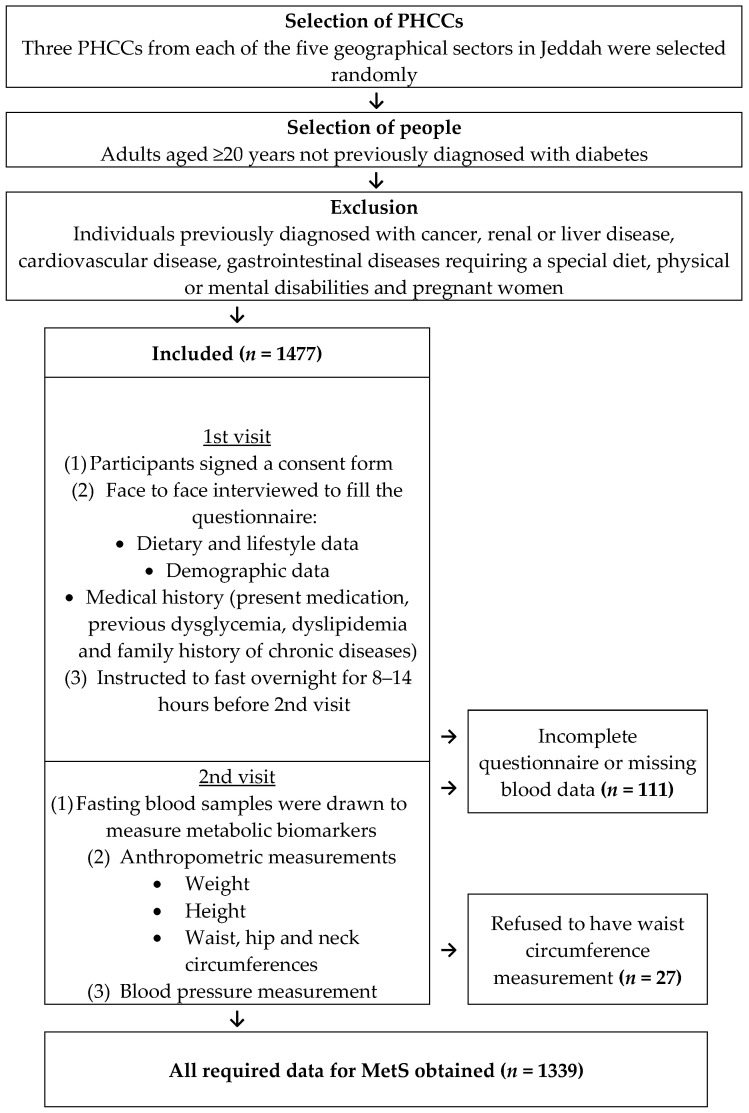
Recruitment flow chart.

By the end of February 2017, 1477 adults had been enrolled in the study (Figure 1). Data collection was completed for 1339 participants. The absence of data for the remaining participants was attributed to missing, hemolyzed, broken, or unlabeled blood samples, which occurred randomly and are unlikely to impact the study’s validity. After conducting biochemical tests, it was determined that 212 individuals (16%) (118 men and 94 women) had metabolic syndrome (Table 1).

### 3.1. Association between MetS and Anthropometric Measurements

Table 1 illustrates the demographic, clinical, and anthropometric profiles of the study’s male and female participants. Notable demographic differences were observed between the non-metabolic syndrome (non-MetS) and metabolic syndrome (MetS) groups. The MetS group exhibited notably older average age, a higher proportion of married individuals, more people with children, and participants with lower education and income levels with statistical significance of *p* ≤ 0.01 (Table 1).

In terms of anthropometric data, the MetS group had significantly higher average body mass index (BMI), weight, body fat percentage, neck circumference (NC), waist circumference (WC), hip circumference (HC), and higher waist-to-hip and waist-to-height ratios with *p* < 0.001 (Table 1). Furthermore, individuals with MetS showed significantly elevated average systolic and diastolic blood pressure (BP), as well as higher levels of total cholesterol (TC), triglycerides (TG), low-density lipoprotein cholesterol (LDL-C), hemoglobin A1C (HbA1C), and both fasting and 1 h plasma glucose levels, along with lower high-density lipoprotein cholesterol (HDL-C), compared to those without MetS with *p* < 0.001 (Table 1). The only lifestyle factor that was significantly associated with MetS was low physical activity, as MetS was less prevalent in people who reported engaging in physical activity of at least 30 min per day for at least five days per week (13.6% vs. 17.6%, *p* < 0.05 at least, Table 1). There was no significant difference in daily intake of any dietary items between the non-MetS and MetS groups, except for the energy drinks, with the MetS group having a significantly lower means of daily intake (*p* < 0.01 at least, Table 1) and hibiscus and cinnamon drinks with the MetS group having a significantly higher means of daily intake (*p* < 0.05 at least, Table 1).

### 3.2. Prevalence of MetS and Its Components in Age and Sex Groups

#### 3.2.1. The Overall Prevalence of MetS by Age and Sex

**Figure 2:**
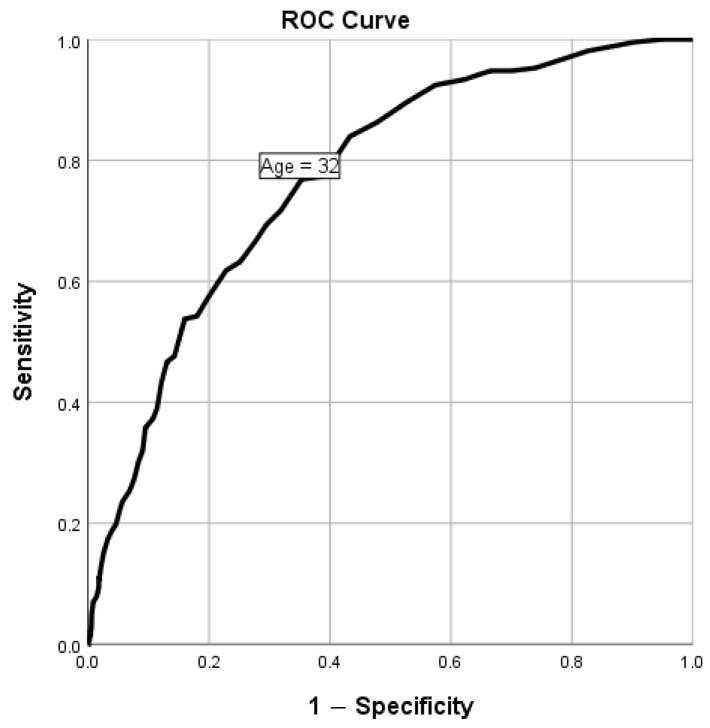
The discriminative power of age for MetS using the receiver operating characteristic curve.

There was no association between sex and MetS in general (Table 1). The overall prevalence of MetS was found to be significantly increased with age, as MetS was prevalent in 0% of people < 20 years, 5.5% of 20–29 years, 17.2% of 30–39 years, 37% of 40–49 years, 34.8% of 50–59 years, 54.3% of 60–69 years, and 50% of those 70+ years of age (*p* < 0.001, Figure 2). The optimal cut-off level of age in predicting MetS was 32 years (AUC (95% CI): 0.772 (0.739, 0.804), *p* < 0.001; Figure 2), with a sensitivity of 76.9% and sensitivity of 64.7%. This was determined to enable categorizing participants into two age groups to investigate the predictors of MetS in those groups separately.

**Figure 3:**
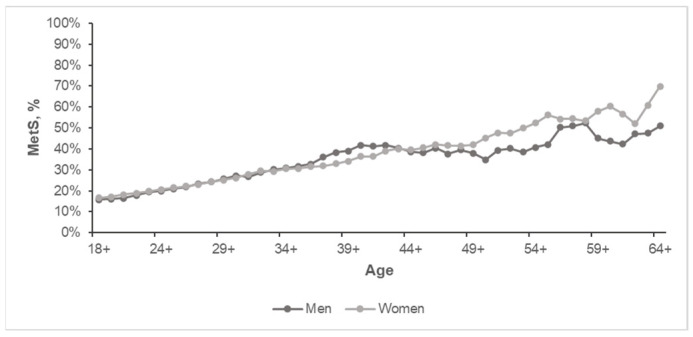
Cumulative prevalence of MetS for the population aged 18+ to 63+ years.

**Figure 4:**
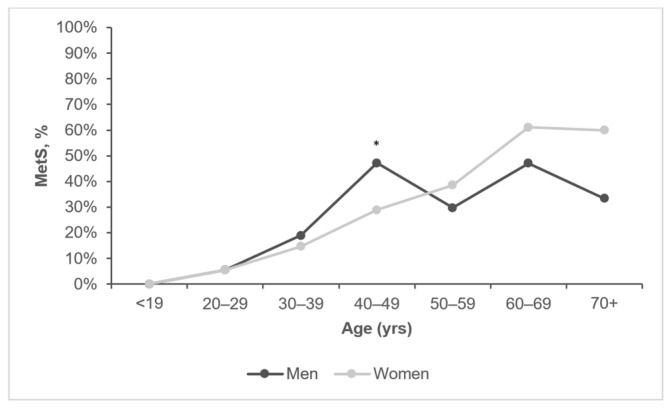
Prevalence of MetS in different age and sex groups. Men n = 24, 379, 228, 72, 37, 17, and 3, respectively. Women n = 33, 237, 144, 90, 52, 18, and 5, respectively. * denotes a significant difference in MetS % between sex groups using the chi-square test.

The cumulative prevalence of MetS with age (Figure 3) showed that the trend of its increase differs in sex groups after the age of 35 years. The stratification of study participants by ten-year age groups and sex showed that men sustained a higher prevalence of MetS than women until 50 years of age. The prevalence of MetS in men peaked at 40–49 years of age (47.2%), where it became significantly higher than in women (28.9%) of the similar age group (*p* < 0.05, Figure 4). MetS prevalence in men fluctuated after 50 years of age as it dropped to 29.7% in men aged 50–59 years and then increased to 47.1% in those aged 60–69 years. Women sustained a steady increase in the prevalence of MetS until the age of 60–69 years, where it peaked (61.1%) (Figure 4). Men in this age group had a 47.1% prevalence of MetS, but this was not significantly lower than women.

#### 3.2.2. Prevalence of MetS Components by Age and Sex

**Figure 5:**
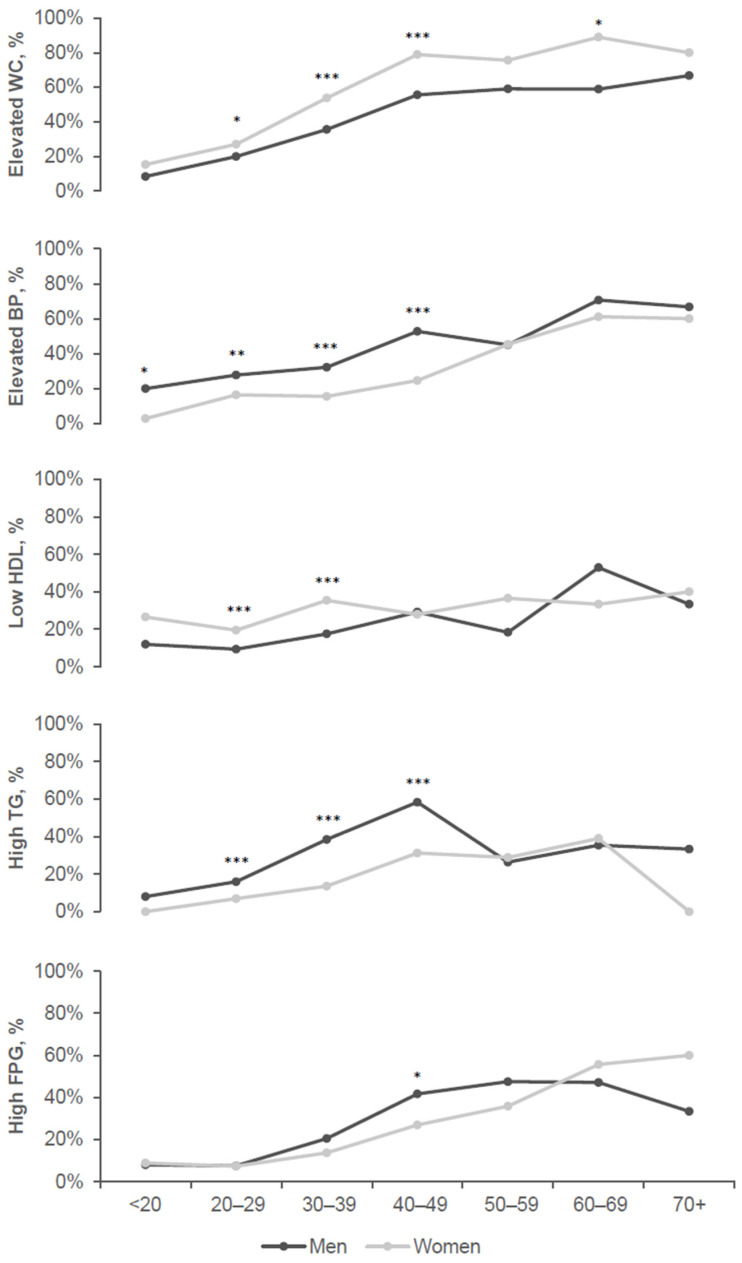
Prevalence of MetS components in different age and sex groups. * denotes significant difference in MetS % between sex groups (*p* < 0.05), ** (*p* < 0.01) and *** (*p* < 0.001).

The overall prevalence of elevated WC, elevated BP, low HDL-C, high TG, and high FPG was 38.1%, 28.3%, 20.6%, 22.1%, and 17.2%, respectively. The prevalence of MetS components in different sex groups in <32 and 32+ years adults is displayed in (Table 2), and all of them were significantly more prevalent in 32+ years adults compared with <32 years in both sex groups (*p* < 0.001 for all components except for low HDL-C in women *p* < 0.02). The prevalence of MetS components in different ages by 10 years and sex groups is displayed in Figure 5.

The prevalence of MetS components differed between age and sex groups. In adults <32 years of age, elevated BP was the most prevalent component in men (28.2%), followed by elevated WC (21.7%) and high TG (17.2%), whereas in women, elevated WC was the most prevalent (26.7%) followed by low HDL-C (23.5%) and then elevated BP (15%). In adults 32+ years of age, elevated WC was the most prevalent component in men (45.8%) followed by high TG (43.9%) and elevated BP (41.5%), whereas, in women, elevated WC was the most prevalent (71.6%) followed by low HDL-C (32.4%) and then elevated BP (29.4%).

Elevated WC was more prevalent in women than men of all ages, which was significant in 32+ years adults (*p* < 0.001, Table 2). It peaked in women at the age of 60–69 years (88.9%) and in men at the age of 70+ (66.7%) (Figure 5). Low HDL-C was also significantly more prevalent in women than men in both <32 years (23.5% and 10.6%, respectively, *p* < 0.001; Table 2) and 32+ years adults (32.4% and 22.8%, respectively, *p* < 0.02; Table 2). The prevalence of low HDL-C increased by age until 70+ years in women, whereas in men, it peaked at 60–69 years (Figure 5). Elevated BP was significantly more prevalent in men than women in both <32 years (28.2% and 15%, respectively, *p* < 0.001; Table 2) and 32+ years adults (41.5% and 29.4%, respectively, *p* < 0.01; Table 2). The prevalence of elevated BP peaked at 60–69 years in both sex groups (Figure 5). High TG was also significantly more prevalent in men than women in both <32 years (17.2% and 6.2%, respectively, *p* < 0.001; Table 2) and 32+ years adults (43.9% and 25.4%, respectively, *p* < 0.01; Table 2). The prevalence of high TG peaked in men at 40–49 years (58.3%) and dropped to 26.3% at age 50–59 (Figure 5). There were no differences in the prevalence of high FPG between sex groups when analyzed separately in <32 years and 32+ years adults. However, the analysis in separated age groups by ten years showed that at age 40–49 years, high FPG was significantly more prevalent in men (46.7%) than women (26.9%) (*p* < 0.05, Figure 5).

**Figure 6:**
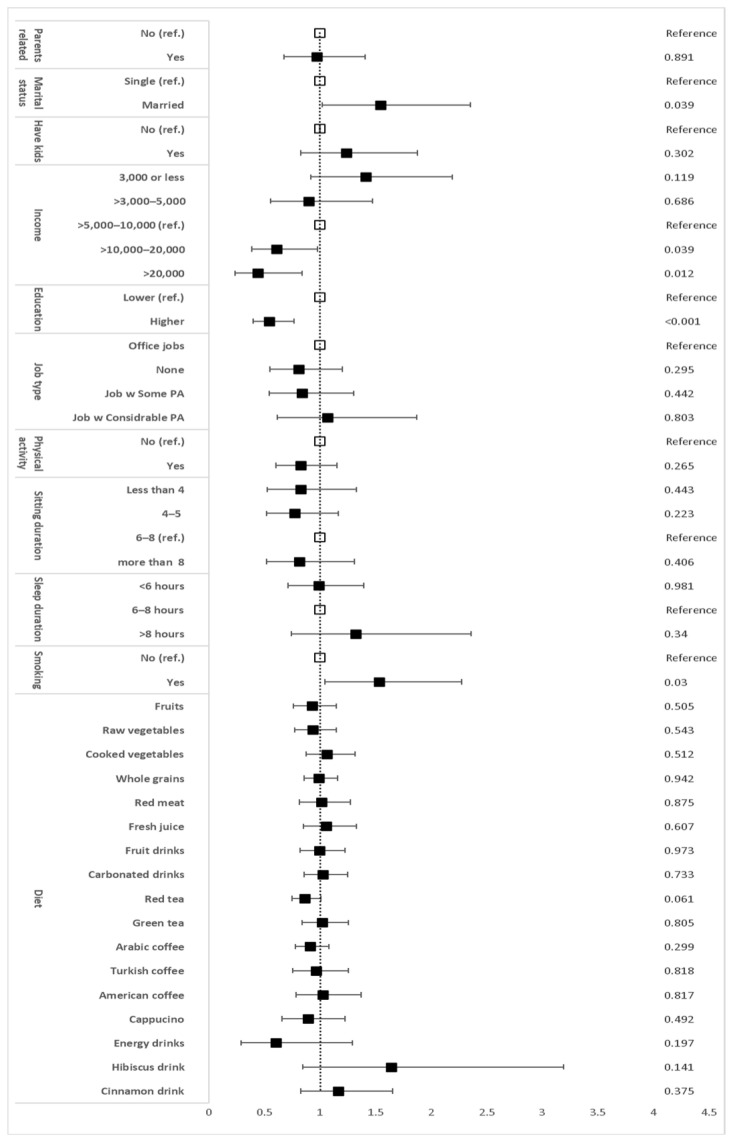
Univariate logistic regression and *p*-values of demographic, lifestyle, and dietary factors with MetS. Data are prevalence ratio. Error bars represent the 95% confidence intervals (CIs). *p*-values are shown in the left column. The clear squares indicate the reference group. The black squares indicate the groups that are being compared with the reference group.

**Figure 7:**
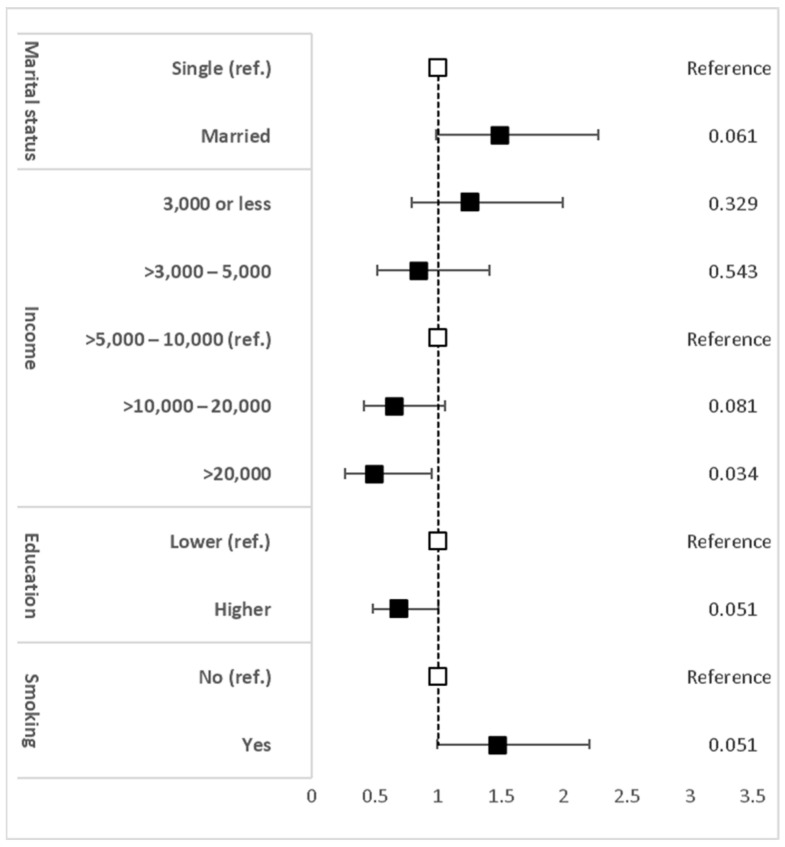
Multivariate logistic regression and *p*-values of marital status, income (Saudi Riyal, SAR), education, and smoking with MetS after adjustment for age and gender. Data are prevalence ratio. Error bars represent the 95% confidence intervals (CIs). *p*-values are shown in the left column. The clear squares indicate the reference group. The black squares indicate the groups that are being compared with the reference group.

The PR for demographic, lifestyle and dietary factors associated with MetS are illustrated in Figure 6. High income of SAR 10,000–20,000 and >SAR 20,000 and higher education were significantly associated with a lower probability of MetS compared with a medium income of SAR 5000–10,000 and lower education (SAR 10,000–20,000 PR = 0.613, *p* < 0.05, >SAR 20,000 PR = 0.445, *p* < 0.02, and higher education PR = 0.555, *p* < 0.001; Figure 6). Marriage and smoking were significantly associated with MetS (married PR = 1.552, *p* < 0.05, and smoking PR = 1.54, *p* < 0.05; Figure 6). After adjusting for age and sex, the multivariate logistic regression for these four factors with MetS showed that only a high income of >SAR 20,000 was significantly associated with a lower probability of MetS (PR = 0.504, *p* = 0.034; Figure 7). It also showed that the high income of SAR 10,000–20,000 and higher education tended to be significantly associated with a lower probability of MetS (PR = 0.657 and PR = 0.694, respectively, *p* = 0.081 and 0.051, respectively; Figure 7), whereas being married and smoking tended towards being significantly associated with an increased risk of MetS (PR = 1.493 and PR = 1.481, respectively, *p* = 0.061 and 0.051, respectively; Figure 7).

## 4. Discussion

Dietary and other lifestyle modifications have been reported to be successful in managing MetS in different populations [24,25,39,40,41,42]. In view of the high prevalence of this disorder in Saudi Arabia [14,43] and its strong association with increased risk of diabetes and CVD [1,2,4], investigating factors associated with the condition is highly required to develop an evidence-based, local management program. Therefore, in this study, we aimed to investigate the demographic, dietary, and other lifestyle practices associated with an increased risk of MetS in our Saudi population. The city of Jeddah was chosen, as outlined earlier [23], because its population is of mixed ethnicities with a varied mixture of dietary and other lifestyle habits and practices, reflecting the country’s multi-ethnic nature. Excluding individuals with previously diagnosed diabetes and other chronic health conditions requiring pharmacotherapy helps to ensure that the measured biochemical variables were not modulated by treatment.

Furthermore, this approach ensured that reported lifestyle and dietary habits were usually practiced and not modified due to medical advice after diagnosis. As for those found to have dyslipidemia or hypertension, they were mostly not previously diagnosed. Hence, very few people were taking medications regularly.

We found that MetS was diagnosed in 16% of the studied population, with all the studied anthropometric and biochemical measurements and some of the demographic and lifestyle characteristics significantly different between people with and those without MetS. However, there was no association between the intake of fruits and vegetables, whole grains, red meat, juices and drinks, red and green tea, all types of coffee, and energy drinks with MetS. In addition, there was no association between sex and MetS in general, and age was found to be its strongest predictor. The increasing prevalence of MetS with advancing age in men and women has been long reported [44,45,46,47]. The optimal cut-off age to predict MetS was 32 years. Moreover, the prevalence of the components of MetS differed among age and sex groups. In adults < 32 years of age, elevated BP was the most prevalent component in men (28.2%), whereas in women, elevated WC was the most prevalent one (26.7%). In adults 32+ years of age, elevated WC was the most prevalent component in both men (45.8%) and women (71.6%).

After adjusting for age and gender in the multivariate logistic regression, a high income of >SAR 20,000 was significantly associated with a low risk of MetS. In contrast, an income of SAR 10,000–20,000 and higher education only tended towards being significantly associated with a low risk of MetS. On the other hand, being married and a smoker tended to be significantly associated with an increased risk of MetS.

Earlier studies on Saudi and other populations’ prevalence of MetS have reported age and sex dependency, increasing with age with higher prevalence in women [8,44,48,49,50]. On the other hand, the prevalence of MetS in Korea was reported to be higher in men (40%) than women (26.2%) in 2020 [51]. The U.S. NHANES data collected from 2015 to 2016 showed that the overall prevalence of MetS was not different between men and women (35.1% vs. 34.3%, respectively) [52]. In an earlier study by our group in the same location and employing the same methodology, a higher prevalence of 21.9% was found, with age and sex being the strongest predictors, and the syndrome is more prevalent among women [14], unlike the present study which found no such association with sex. The difference between the two studies could be attributed to the radical changes in lifestyle habits over time between the two studies. Due to the recently implemented state policy of empowering women, a higher percentage of women are employed in various sectors, and gyms for women have been opened, hence increasing the likelihood of increased physical activity, as well as improved regulation of sleeping hours and eating patterns, hence decreasing the traditional risk factors for MetS. Like our findings, a study conducted in Riyadh found no significant difference between men and women in the prevalence of MetS [12].

Similar to our findings, several earlier studies have reported that MetS is significantly associated with adult marital status [53], particularly in married women [54,55]. In a Saudi study with a larger sample size than ours, a higher prevalence of MetS among married men but not women was reported [56]. We did not investigate sex differences when analyzing the effect of various characteristics due to the smaller sample size.

In partial agreement with our results, previous studies in other countries reported an inverse association of MetS with educational level in both sexes and with income in women only [57] and in both sexes [57]. In contrast, another study reported a positive association of MetS with higher income and education among men but not in women [58], while another study reported a higher prevalence of MetS in men with high incomes [59]. Similarly, a Tunisian study reported a higher occurrence of MetS among adults with a higher socioeconomic status [60]. Furthermore, the earlier Saudi study showed no association between education and the prevalence of MetS and a direct association with higher income in men only [56]. The difference in results between our study and the mentioned Saudi study could be due to various reasons, including the health status of included people (people with diabetes were not included in our study), differences in the percentage of other ethnicities (Riyadh with more people from Arabian tribes versus Jeddah with a multi-ethnic population), use of different criteria to define MetS (we used the consensus definition as mentioned in the methods section, while they used NCEP ATP III diagnostic criteria), and finally the period of data collection was earlier in the Riyadh study (2009 vs. 2016–2017 in our study).

Smoking has been reported to be associated with increased risk of various components of metabolic syndrome and hence could lead to the development of the full syndrome by multiple mechanisms [61]. Indeed, previous studies have reported that smoking is associated with metabolic abnormalities and increases the risk of MetS [62]. A more recent Korean study confirmed the association between MetS and smoking, reporting that cigarette smokers had a 2.4-fold greater risk of the full syndrome compared with non-cigarette smokers, as well as an increased risk of several components of the syndrome, including a 2.6-fold greater risk of hypertriglyceridemia and a three-fold greater risk of low HDL-C [63].

In accordance with our results, a meta-analysis of 13 prospective studies found that active smoking was associated with an increased risk of MetS. However, further stratified analysis by sex found that male smokers seemed to have a substantially greater metabolic syndrome risk (pooled RR 1.34, 95% CI: 1.20–1.50) compared with female smokers (pooled RR 0.85, 95% CI: 0.60–1.21), with the difference being statistically significant in meta-regression analysis (58). In an earlier study by our group, smoking was found to be associated with an increased risk of dyslipidemia (a component of metabolic syndrome) in men (PR 1.41, 95% CI: 1.00, 1.99, *p* = 0.043) [64].

In addition to smoking, the development of MetS has been reported to be influenced by dietary intake, alcohol consumption, and physical exercise [65]. Lifestyle practices, unhealthy dietary practices, and low levels of physical activity can cause an increase in body fat and insulin resistance, which could alter biochemical parameters, such as the lipids profile and glycemic indicators, towards a profile characteristic of MetS [66]. In this study, we found no association between the level of physical activity or sleeping habits and MetS, even though in the earlier study by our group mentioned above, sleep duration < 6 h was associated with an increased risk in men of one of the MetS components, namely dyslipidemia (PR: 1.573, 95% CI: 1.14, 2.18, *p* = 0.006) [67].

Various studies have investigated the association between dietary patterns and MetS, such as fruit and dairy dietary patterns in the Mediterranean Diet [68,69]. On the other hand, some studies investigated the influence of eating habits, concluding that some habits can lead to the development and progression of MetS [70,71,72,73]. We did not investigate dietary patterns or habits in our study. Instead, we focused on some food types that have been shown to be associated with the risk of the syndrome or its components, such as fruit, vegetables, red meat, whole grains, sweetened drinks, herbal drinks, tea, and coffee. Higher intake of whole grains has been reported to be associated with decreased risk of MetS, while highly processed cereals with a high glycemic index (GI) were found to be associated with higher risk [74], while no such association was detected in our study. The difference between our findings and the earlier study could be due to the combined effect of other dietary components or lifestyle factors.

The association of MetS with the consumption of fruit and/or vegetables was investigated in different populations. Two meta-analyses found that fruit and/or vegetable consumption may be inversely associated with the risk of MetS [75,76]. On the other hand, another meta-analysis, which also conducted a dose–response analysis, found a minimal decrease in risk of MetS (PR 0.97, 95% CI: 0.95, 0.99) for an increase of 100 g/d in fruit consumption. In contrast, an increase of 100 g/d in vegetable consumption was not associated with a reduction in MetS [77], which could explain the reason for not detecting any association between fruit and vegetable intake in our present study. Furthermore, in two earlier studies, we investigated the association of fruit and vegetable intake with components of MetS and found that, in women, an increased intake of fresh vegetables was associated with increased risk of dyslipidemia (PR: 2.07, 95% CI: 1.09, 3.94, *p* = 0.026), which could be attributed to added salad dressing [67], while an intake of fresh juice of one to four portions per week and five portions or more were found to be associated with decreased risk of dysglycemia (PR 0.603 (95% CI: 0.369, 0.985; *p* = 0.043) and PR 0.511 (95% CI: 0.279, 0.935; *p* = 0.029), respectively) compared with women who did not drink fresh juice [78], adding another reason for not finding an effect of fruit and vegetable intake on the risk of MetS in our present study.

The association between various meat intakes and the risk of MetS was assessed in a meta-analysis of observational studies, concluding that total, red, and processed meat consumption was positively associated with metabolic syndrome. In contrast, the intake of white meat was inversely associated with the syndrome [79]. However, the effects were very slight, with the pooled RR for MetS of the highest versus lowest category of meat intake being 1.14 (95% CI: 1.05, 1.23) for total meat, 1.33 (95% CI: 1.01, 1.74) for red meat, 1.35 (95% CI: 1.18, 1.54) for processed meat, and 0.86 (95% CI: 0.76, 0.97) for white meat. Moreover, in our earlier study [78], a weekly intake of five portions or more of red meat was found to be associated with decreased odds of having dysglycemia in men with PR 0.444 (95% CI: 0.223, 0.881; *p* = 0.02) following adjustment for age, BMI, and waist circumference. Dysglycemia in the case of the aforementioned study was identified by more than normal levels of FBG, and/or 1 h blood glucose following glucose load, and/or glycated hemoglobin, unlike the present study, which depended only on FBG to define dysglycemia as required by the consensus definition of MetS [5]. The minimal effects noted in the meta-analysis [79] (Kin and Je), as well as the decreased risk of dysglycemia found in our earlier study [78], could explain the lack of association noted in our present study.

The association between the consumption of sugar-sweetened beverages and MetS was investigated in a meta-analysis, concluding that higher consumption of these drinks was associated with the development of the syndrome, and the effect is more pronounced than that which was noted for fruit and vegetables or red meat (the pooled RR was 1.20, 95% CI: 1.02–1.42) [80]. However, like our findings, in a cross-sectional study on Iranian adults, no significant associations were found between the intake of sugary beverages and the prevalence of metabolic syndrome in either gender [81].

Hibiscus infusion is a very popular drink in many parts of the world as part of traditional medicine due to the presence of bioactive compounds that exert antioxidant and anti-inflammatory effects, which contribute to reducing CVD risk markers [82]. However, unlike our study, published studies have focused on its therapeutic uses in treating various metabolic dysregulations and clinical conditions [83], not on its preventative potential or its association with these disorders. The data from human trials support hibiscus’s use in moderating BP [84,85] and blood lipids [86]. A recent review of animal studies and clinical trials to assess hibiscus’s effectiveness for treating MetS biomarkers improved blood glucose, TC, HDL-C, TG, and BP [87]. It is a common practice in this region to consume hibiscus infusion to manage these metabolic dysregulations and to control high BP. However, our sample of the population was non-diabetic, and they were mostly unaware of their health status; hence, only a few individuals consumed five or more portions/week, and after adjusting for age and sex, no association was detected with MetS, even though in our earlier study the intake of five times or more per week of hibiscus drink was associated with increased odds of having dysglycemia in women, PR 5.551 (95% CI: 1.576, 19.55, *p* = 0.008) compared with women not using such a drink [78].

The dried flowers and leaves of the plant Camellia sinensis are commonly used to prepare an infusion, namely tea, which is a popular global beverage. Based on the oxidation degree of the plant, white, green, yellow, oolong, black, and pu-ehr are produced [88]. Black and green tea are the two most consumed in our region; hence, both were included in this study. The consumption of both forms of tea has been reported to decrease the risk of developing several chronic disorders and diseases, including obesity, dyslipidemia, hypertension, MetS, and diabetes [89,90,91,92,93], due to its thermogenic, cholesterol-lowering, antihypertensive, antioxidant, anti-inflammatory, and neuroprotective effects [94,95]. However, some studies reported that higher tea consumption was associated with an increased risk of MetS [96,97].

Furthermore, other studies reported no association between tea consumption and risk of the syndrome [98,99], like our findings in this study. The yet unresolved association could be due to differences in research methodologies, studied populations, and, more importantly, differences in the preparation of the beverage and the water used for the preparation and the addition of other ingredients, including sugar. More studies using a cohort design and including all possible factors affecting the final content of the beverage are needed to clarify the situation, especially since one of our earlier studies reported that high tea consumption was associated with hypothyroidism due to its high fluoride content [100].

Coffee is another popular beverage in our region and among our studied population. Several studies reported that coffee consumption may exert beneficial metabolic effects, and long-term coffee consumption has been associated with a decreased risk of type 2 diabetes [101,102] and cardiovascular disease [103]. Moreover, the association between coffee consumption and the risk of MetS has been investigated in various studies, and an inverse association between high habitual intakes of more than three cups of coffee per day and MetS has been reported [104]. Furthermore, two meta-analyses found an inverse relationship between MetS and coffee consumption [105,106]. However, observational studies showed that the acute effects of coffee differ from the long-term effects [107]. An example is noted when coffee consumption showed no substantial effects on the risk of hypertension in a meta-analysis of observational studies, but an acute rise in blood pressure was reported in a meta-analysis of randomized controlled trials [106,108]. Furthermore, the concentrations of coffee chemical components can be influenced by agricultural factors (e.g., species and variety of plant and cultivation methods) and preparation approaches (e.g., roasting, blending, and brewing) [109,110]. In this study, no association between the intake of coffee and MetS was found, in contrast to our findings in our earlier studies on the same population where an association was found with some components of MetS and an intake of ≥five portions/week of Turkish coffee was found to be associated with decreased odds of having dysglycemia [78], while the same intake was associated with increased risk of dyslipidemia in men [67].

Finding no association between all investigated dietary components and lifestyle characteristics with MetS in women was interesting. This could be due to the minimal effect of each variable, so it could not be detected with the sample size in our study. On the other hand, the combined effect of one variable might cancel the effect of another, leading to no net effect. A future study using the same data and employing clustering of factors in different ways is planned to clarify the situation further.

Our study has several points of strength as well as a few limitations. The first and main limitation is the use of cross-sectional design, which does not adequately allow inferences about cause and effect but can only suggest associations. Furthermore, it does not allow differentiating between the acute and chronic effects of the studied components. However, this design is commonly used to help direct future, more focused cohort studies. Another limitation is expected errors in reporting while collecting data using a questionnaire involving self-reporting of dietary and lifestyle practices. This kind of error is common in all similar papers relying on questionnaires to collect information. Therefore, to minimize these errors, we conducted face-to-face interviews with trained data collectors who used previously constructed prompting questions to increase the reliability of given responses. Finally, due to time and budget limitations, our sample size was relatively small. Despite this, several associations were detected.

The first point of strength in our study is that it is the first in our region to include various lifestyle and dietary practices. Another point of strength is the avoidance of bias in sample selection by random selection of PHC and included volunteers. In addition, data collection was carried out using standardized methods by well-trained data collectors. Furthermore, all biochemical analyses were performed in one accredited lab to avoid variations leading to misclassification.

## 5. Conclusions

In conclusion, MetS was detected in 16% of the studied population, with no sex difference. Age was its strongest predictor in both men and women. After adjusting for age and sex, only a very high income of >SAR 20,000 was significantly associated with a low risk of MetS, while high education only tended to be associated with a low risk of MetS (Figure 5). On the other hand, being married and a smoker tended to be significantly associated with an increased risk of MetS. Further cohort studies are needed to validate our findings and to provide a better understanding of the factors associated with MetS in the Saudi population.

## Figures and Tables

**Table 1:** Demographic, anthropometric, clinical, biochemical, lifestyle, and dietary characteristics of participants by MetS status.

	Non-MetS *n* = 1127	MetS *n* = 212	*p*-Value
Demographical characteristics			
**Age (years) *mean ± SD***	30.5 ± 10.2	41.7 ± 12.4	<0.001 ^a^
**Sex *n (n%)***			
Men	642 (84.5%)	118 (15.5%)	0.725 ^b^
Women	485 (83.8%)	94 (16.2%)	
**Parents consanguinity *n (n%)***			
No	838 (83.8%)	162 (16.2%)	0.527 ^b^
Yes	289 (85.3%)	50 (14.7%)	
**Marital status *n (n%)***			
Single	570 (93%)	43 (7%)	<0.001 ^b^
Married	557 (76.7%)	169 (23.3%)	
**Have children *n (n%)***			
No	304 (89.4%)	36 (10.6%)	0.002 ^b^
Yes	823 (82.4%)	176 (17.6%)	
**Number of children *mean ± SD***	4 ± 3	5 ± 2	0.228 ^a^
**Educational level *n (n%)***			
Lower education or illiteracy	428 (77%)	128 (23%)	<0.001 ^b^
Higher education	699 (89.3%)	84 (10.7%)	
**Job type *n (n%)***			
None	529 (85.2%)	92 (14.8%)	0.584 ^b^
Office job	210 (82.4%)	45 (17.6%)	
Job with some physical activity	288 (84.7%)	52 (15.3%)	
Job with considerable physical activity	100 (81.3%)	23 (18.7%)	
**Income *n (n%)***			
3000 or less	179 (74.6%)	61 (25.4%)	<0.001 ^b^
>3000–5000	188 (83.2%)	38 (16.8%)	
>5000–10,000	280 (82.8%)	58 (17.2%)	
>10,000–20,000	298 (88.4%)	39 (11.6%)	
>20,000	182 (91.9%)	16 (8.1%)	
**Anthropometric measurements *(mean ± SD)***			
**Weight (kg)**			
Men	78.7 ± 17	97 ± 19.4	<0.001 ^a^
Women	65.8 ± 15.1	82.5 ± 16.3	<0.001 ^a^
**BMI**	26.6 ± 5.7	32.8 ± 5.9	<0.001 ^a^
**Fat (%)**			
Men	25.9 ± 9	33.6 ± 7	<0.001 ^a^
Women	38.6 ± 11.3	46 ± 9.6	<0.001 ^a^
**NC (cm)**			
Men	39 ± 4.1	42.5 ± 3.5	<0.001 ^a^
Women	33.1 ± 3.9	36.4 ± 4.3	<0.001 ^a^
**WC (cm)**			
Men	94.3 ± 14.1	112.4 ± 14	<0.001 ^a^
Women	85.7 ± 15.3	102.4 ± 12.6	<0.001 ^a^
**HC (cm)**			
Men	105.3 ± 12.9	117.4 ± 13.3	<0.001 ^a^
Women	103.3 ± 12.9	115.8 ± 13.1	<0.001 ^a^
**WC to HC ratio**			
Men	0.89 ± 0.08	0.96 ± 0.06	<0.001 ^a^
Women	0.83 ± 0.09	0.89 ± 0.08	<0.001 ^a^
WC to height ratio	0.55 ± 0.09	0.65 ± 0.08	<0.001 ^a^
**Clinical measurements *mean ± SD***			
BP-Systolic	115.7 ± 13.1	129.2 ± 19.5	<0.001 ^a^
BP-Diastolic	71.8 ± 11.1	80.6 ± 12.3	<0.001 ^a^
**Biochemical measurements *mean ± SD***			
Serum TC (mmol/L)	4.8 ± 0.9	5.1 ± 1	<0.001 ^a^
Serum HDL-C (mmol/L)			
Men	1.3 ± 0.2	1.1 ± 0.2	<0.001 ^a^
Women	1.5 ± 0.3	1.3 ± 0.2	<0.001 ^a^
Serum LDL-C (mmol/L)	3.2 ± 0.8	3.5 ± 0.9	<0.001 ^a^
Serum TG (mmol/L)	1.1 ± 0.7	2.1 ± 1.1	<0.001 ^a^
HbA1c %	5.2 ± 0.5	5.7 ± 1	<0.001 ^a^
Plasma glucose (0 h)	4.3 ± 0.9	5.2 ± 1.7	<0.001 ^a^
Plasma glucose (1 h)	6.4 ± 2.1	8.4 ± 3	<0.001 ^a^
**Lifestyle factors**			
**Physical activity of at least 30 min per day for at least 5 days per week *n (n%)***			
No	618 (82.4%)	132 (17.6%)	0.046 ^b^
Yes	509 (86.4%)	80 (13.6%)	
Sitting h/day *n (n%)*			
<4	207 (82.8%)	43 (17.2%)	0.868 ^b^
4–5	348 (84.9%)	62 (15.1%)	
6–8	350 (83.7%)	68 (16.3%)	
8+	222 (85.1%)	39 (14.9%)	
**Sleep duration** ***n (n%)***			
<6 h	432 (83.1%)	88 (16.9%)	0.657 ^b^
6–8 h	590 (85%)	104 (15%)	
>8 h	105 (84%)	20 (16%)	
**Smoking habits** ***n (n%)***			
Non-smoker	858 (85%)	152 (15%)	0.243 ^b^
Smoker	230 (81%)	54 (19%)	
Previous smoker	39 (86.7%)	6 (13.3%)	
**Dietary items (portion/day) *mean ± SD***			
Fruits	0.66 ± 0.81	0.7 ± 0.78	0.524 ^a^
Raw vegetables	0.79 ± 0.83	0.82 ± 0.77	0.63 ^a^
Cooked vegetables	0.65 ± 0.77	0.73 ± 0.75	0.135 ^a^
Whole grains	1.17 ± 1.05	1.23 ± 1.12	0.467 ^a^
Red meat	0.57 ± 0.71	0.57 ± 0.71	0.979 ^a^
Fresh juice	0.47 ± 0.72	0.49 ± 0.65	0.769 ^a^
Fruit drinks	0.57 ± 0.83	0.51 ± 0.75	0.344 ^a^
Carbonated drinks	0.62 ± 0.91	0.51 ± 0.85	0.127 ^a^
Red tea	0.96 ± 1.1	1.04 ± 1.12	0.3 ^a^
Green tea	0.37 ± 0.72	0.49 ± 0.83	0.063 ^a^
Arabic coffee	0.63 ± 0.99	0.63 ± 0.97	0.99 ^a^
Turkish coffee	0.24 ± 0.62	0.23 ± 0.6	0.82 ^a^
American coffee	0.16 ± 0.55	0.17 ± 0.57	0.932 ^a^
Cappuccino	0.26 ± 0.59	0.19 ± 0.49	0.051 ^a^
Energy drinks	0.12 ± 0.43	0.04 ± 0.17	0.009 ^a^
Hibiscus drink	0.03 ± 0.18	0.08 ± 0.29	0.033 ^a^
Cinnamon drink	0.08 ± 0.37	0.15 ± 0.44	0.027 ^a^

NC = neck circumference; WC = waist circumference; HC = hip circumference; BP = blood pressure; TC = total cholesterol; HDL-C = high-density lipoprotein cholesterol; LDL-C = low-density lipoprotein cholesterol, TG = triglycerides, HbA1c = glycated hemoglobin. *p*-values that reached statistical significance are highlighted in bold. ^a^ *p*-value was derived using an independent *t*-test or the Mann–Whitney U test when the normality assumption was not met for comparing mean values between the groups with and without metabolic syndrome (MetS). ^b^ *p*-value was derived using the chi-square test for comparing proportions between the two groups.

**Table 2:** Prevalence of MetS and its components in different sex groups in people <32 years and 32+ years of age.

	Age < 32					Age 32+				
	Men *n* = 471		Women *n* = 307		*p*-Value ^b^	Men *n* = 289		Women *n* = 272		*p*-Value ^b^
	*n* (%)	Mean ± SD	*n* (%)	Mean ± SD		*n* (%)	Mean ± SD	*n* (%)	Mean ± SD	
**WC ^**										
**Normal WC**	369 (78.3)	87.4 ± 9.4	225 (73.3)	75.2 ± 7.4	**<0.001**	156 (54.2)	92.7 ± 7.5	77 (28.4)	78.7 ± 7.8	**<0.001**
**Elevated WC**	102 (21.7)	115.8 ± 12.1	82 (26.7)	100 ± 10.2	**<0.001**	132 (45.8)	115 ± 10	194 (71.6)	103 ± 12	**<0.001**
***p*** **-value ^a^**	0.105					**<0.001**				
**BP ^**										
**Normal BP**	338 (71.8)	SBP: 115.1 ± 8.3	261 (85)	SBP: 107 ± 10	**<0.001**	169 (58.5)	SBP: 116 ± 7	192 (70.6)	SBP: 109 ± 10	**<0.001**
		DBP: 69.8 ± 9.2		DBP: 66.4 ± 8.4	**<0.001**		DBP: 72.9 ± 8		DBP: 67.4 ± 8.3	**<0.001**
**Elevated BP**	133 (28.2)	SBP: 131.2 ± 11.3	46 (15)	SBP: 127 ± 15	0.061	120 (41.5)	SBP: 136 ± 17	80 (29.4)	SBP: 134 ± 19	0.461
		DBP: 82.8 ± 11.3		DBP: 86.2 ± 9.6	0.054		DBP: 85 ± 10.4		DBP: 83.8 ± 11.8	0.462
***p*** **-value ^a^**	**<0.001**					**0.003**				
**HDL-C ^**										
**Normal HDL-C**	420 (89.4)	1.3 ± 0.2	235 (76.5)	1.6 ± 0.2	**<0.001**	223 (77.2)	1.3 ± 0.2	184 (67.6)	1.6 ± 0.2	**<0.001**
**Low HDL-C**	50 (10.6)	0.9 ± 0.1	72 (23.5)	1.1 ± 0.2	**<0.001**	66 (22.8)	1 ± 0.2	88 (32.4)	1.2 ± 0.2	**<0.001**
***p*** **-value ^a^**	**<0.001**					**0.012**				
**TG ^**										
**Normal TG**	389 (82.8)	0.94 ± 0.32	288 (93.8)	0.79 ± 0.27	**<0.001**	162 (56.1)	1.1 ± 0.33	203 (74.6)	1 ± 0.33	**0.007**
**High TG**	81 (17.2)	2.66 ± 1.24	19 (6.2)	1.98 ± 0.73	**0.024**	127 (43.9)	2.68 ± 1.04	69 (25.4)	2.03 ± 0.88	**<0.001**
***p*** **-value ^a^**	**<0.001**					**<0.001**				
**FPG ^**										
**Normal FPG**	427 (90.7)	4.2 ± 0.5	282 (91.9)	4.1 ± 0.5	0.82	198 (68.5)	4.4 ± 0.6	202 (74.3)	4.3 ± 0.6	0.097
**High FPG**	44 (9.3)	5.1 ± 1.3	25 (8.1)	5 ± 0.9	0.598	91 (31.5)	6 ± 2.7	70 (25.7)	5.4 ± 2.1	0.16
***p*** **-value ^a^**	0.565					0.132				
**MetS ^**										
**No MetS**	436 (92.6)		293 (95.4)			206 (71.3)		192 (70.6)		
**MetS**	35 (7.4)		14 (4.6)			83 (28.7)		80 (29.4)		
*p*-value ^a^	0.107					0.857				

^a^ *p*-value was obtained to compare the proportions between the two sex groups in each age group using the chi-square test. ^b^ *p*-value was obtained to compare the means of the two groups of people with MetS and without MetS using an independent *t*-test. ^ all components were significantly more prevalent in 32+ years adults compared with <32 years in both sex groups (*p* < 0.001 for all components except for low HDL in woman *p* = 0.014). Statistically significant *p*-values are shown in bold font.

## Data Availability

The datasets generated and/or analyzed during the current study are available in the King Abdulaziz University repository, http://www.kau.edu.sa/GetFile.aspx?id=310042&fn=AnthropometricandMetSdataavailabilitycheck.rar.
