# Correction: Eldakhakhny et al. Prevalence and Factors Associated with Metabolic Syndrome Among Non-Diabetic Saudi Adults: A Cross-Sectional Study. *Biomedicines* 2023, *11*, 3242

**DOI:** 10.3390/biomedicines14040932

**Published:** 2026-04-20

**Authors:** Basmah Eldakhakhny, Sumia Enani, Hanan Jambi, Ghada Ajabnoor, Jawaher Al-Ahmadi, Rajaa Al-Raddadi, Lubna Alsheikh, Wesam H. Abdulaal, Hoda Gad, Anwar Borai, Suhad Bahijri, Jaakko Tuomilehto

**Affiliations:** 1 Department of Clinical Biochemistry, Faculty of Medicine, King Abdulaziz University, Jeddah 22252, Saudi Arabia; gajabnour@kau.edu.sa (G.A.); hjad@kau.edu.sa (H.G.); sb@kau.edu.sa (S.B.); 2 Saudi Diabetes Research Group, King Fahd Medical Research Center, King Abdulaziz University, Jeddah 22252, Saudi Arabia; senani@kau.edu.sa (S.E.); hjambi@kau.edu.sa (H.J.); jalahamade@kau.edu.sa (J.A.-A.); rmsalharbi@kau.edu.sa (R.A.-R.); boraiaa@ngha.med.sa (A.B.); jotuomilehto@gmail.com (J.T.); 3 Food, Nutrition, and Lifestyle Research Unit, King Fahd for Medical Research Center, King Abdulaziz University, Jeddah 22252, Saudi Arabia; 4 Department of Food and Nutrition, Faculty of Human Sciences and Design, King Abdulaziz University, Jeddah 22252, Saudi Arabia; 5 Department of Family Medicine, Faculty of Medicine, King Abdulaziz University, Jeddah 22252, Saudi Arabia; 6 Department of Community Medicine, Faculty of Medicine, King Abdulaziz University, Jeddah 22252, Saudi Arabia; 7 Department of Biochemistry, Faculty of Sciences, King Abdulaziz University, Jeddah 21589, Saudi Arabia; lloo86@hotmail.com (L.A.); whabdulaal@kau.edu.sa (W.H.A.); 8 Medical Biochemistry and Molecular Biology Department, Faculty of Medicine, Alexandria University, Alexandria 21561, Egypt; 9 King Abdullah International Medical Research Center, King Saud Bin Abdulaziz University for Health Sciences, King Abdulaziz Medical City, Jeddah 22384, Saudi Arabia; 10 Department of Public Health, University of Helsinki, FI-00014 Helsinki, Finland; 11 Public Health Promotion Unit, Finnish Institute for Health and Welfare, FI-00271 Helsinki, Finland

## Error in Table

In the original publication [1], there was a mistake in “Table 2. Prevalence of MetS and its components in different sex groups in people <32 years and 32+ years of age” as published. The standard deviation of the diastolic blood pressure (DBP) of the subgroup “Normal BP” in women 32+ years of age should be reported as “8.3” instead of “0.8”. The corrected “Table 2. Prevalence of MetS and its components in different sex groups in people <32 years and 32+ years of age” appears below. The authors state that the scientific conclusions are unaffected. This correction was approved by the Academic Editor. The original publication has also been updated.

## Figures and Tables

**Table 2:** Prevalence of MetS and its components in different sex groups in people <32 years and 32+ years of age.

	Age < 32					Age 32+				
	Men *n* = 471		Women *n* = 307		*p*-Value ^b^	Men *n* = 289		Women *n* = 272		*p*-Value ^b^
	*n* (%)	Mean ± SD	*n* (%)	Mean ± SD		*n* (%)	Mean ± SD	*n* (%)	Mean ± SD	
**WC ^**										
**Normal WC**	369 (78.3)	87.4 ± 9.4	225 (73.3)	75.2 ± 7.4	**<0.001**	156 (54.2)	92.7 ± 7.5	77 (28.4)	78.7 ± 7.8	**<0.001**
**Elevated WC**	102 (21.7)	115.8 ± 12.1	82 (26.7)	100 ± 10.2	**<0.001**	132 (45.8)	115 ± 10	194 (71.6)	103 ± 12	**<0.001**
***p*** **-value ^a^**	0.105					**<0.001**				
**BP ^**										
**Normal BP**	338 (71.8)	SBP: 115.1 ± 8.3	261 (85)	SBP: 107 ± 10	**<0.001**	169 (58.5)	SBP: 116 ± 7	192 (70.6)	SBP: 109 ± 10	**<0.001**
		DBP: 69.8 ± 9.2		DBP: 66.4 ± 8.4	**<0.001**		DBP: 72.9 ± 8		DBP: 67.4 ± 8.3	**<0.001**
**Elevated BP**	133 (28.2)	SBP: 131.2 ± 11.3	46 (15)	SBP: 127 ± 15	0.061	120 (41.5)	SBP: 136 ± 17	80 (29.4)	SBP: 134 ± 19	0.461
		DBP: 82.8 ± 11.3		DBP: 86.2 ± 9.6	0.054		DBP: 85 ± 10.4		DBP: 83.8 ± 11.8	0.462
***p*** **-value ^a^**	**<0.001**					**0.003**				
**HDL-C ^**										
**Normal HDL-C**	420 (89.4)	1.3 ± 0.2	235 (76.5)	1.6 ± 0.2	**<0.001**	223 (77.2)	1.3 ± 0.2	184 (67.6)	1.6 ± 0.2	**<0.001**
**Low HDL-C**	50 (10.6)	0.9 ± 0.1	72 (23.5)	1.1 ± 0.2	**<0.001**	66 (22.8)	1 ± 0.2	88 (32.4)	1.2 ± 0.2	**<0.001**
***p*** **-value ^a^**	**<0.001**					**0.012**				
**TG ^**										
**Normal TG**	389 (82.8)	0.94 ± 0.32	288 (93.8)	0.79 ± 0.27	**<0.001**	162 (56.1)	1.1 ± 0.33	203 (74.6)	1 ± 0.33	**0.007**
**High TG**	81 (17.2)	2.66 ± 1.24	19 (6.2)	1.98 ± 0.73	**0.024**	127 (43.9)	2.68 ± 1.04	69 (25.4)	2.03 ± 0.88	**<0.001**
***p*** **-value ^a^**	**<0.001**					**<0.001**				
**FPG ^**										
**Normal FPG**	427 (90.7)	4.2 ± 0.5	282 (91.9)	4.1 ± 0.5	0.82	198 (68.5)	4.4 ± 0.6	202 (74.3)	4.3 ± 0.6	0.097
**High FPG**	44 (9.3)	5.1 ± 1.3	25 (8.1)	5 ± 0.9	0.598	91 (31.5)	6 ± 2.7	70 (25.7)	5.4 ± 2.1	0.16
***p*** **-value ^a^**	0.565					0.132				
**MetS ^**										
**No MetS**	436 (92.6)		293 (95.4)			206 (71.3)		192 (70.6)		
**MetS**	35 (7.4)		14 (4.6)			83 (28.7)		80 (29.4)		
*p*-value ^a^	0.107					0.857				

^a^ *p*-value was obtained to compare the proportions between the two sex groups in each age group using the chi-square test. ^b^ *p*-value was obtained to compare the means of the two groups of people with MetS and without MetS using an independent *t*-test. ^ all components were significantly more prevalent in 32+ years adults compared with <32 years in both sex groups (*p* < 0.001 for all components except for low HDL in woman *p* = 0.014). Statistically significant *p*-values are shown in bold font.
